# The interaction engine: cuteness selection and the evolution of the interactional base for language

**DOI:** 10.1098/rstb.2021.0108

**Published:** 2022-09-12

**Authors:** Stephen C. Levinson

**Affiliations:** Language and Cognition, Max Planck Institute for Psycholinguistics, Nijmegen, Gelderland, The Netherlands

**Keywords:** interaction engine, language evolution, theory of mind, empathy, cuteness selection, alloparenting

## Abstract

The deep structural diversity of languages suggests that our language capacities are not based on any single template but rather on an underlying ability and motivation for infants to acquire a culturally transmitted system. The hypothesis is that this ability has an interactional base that has discernable precursors in other primates. In this paper, I explore a specific evolutionary route for the most puzzling aspect of this interactional base in humans, namely the development of an empathetic intentional stance. The route involves a generalization of mother–infant interaction patterns to all adults via a process (cuteness selection) analogous to, but distinct from, RA Fisher's runaway sexual selection. This provides a cornerstone for the carrying capacity for language.

This article is part of the theme issue ‘Revisiting the human ‘interaction engine’: comparative approaches to social action coordination’.

## 1. On the puzzle of language evolution

As was much remarked upon in Darwin's time, there is an apparently unbridgeable gulf between human language and the communication systems of other species. Darwin [1] sought to overcome this by invoking distinct phases and mechanisms in the evolution of language (see [2]). The topic of language evolution has always been a controversial area and remains so [3,4], but new palaeontological data and the recovery of ancient DNA have considerably narrowed the scope of the controversy. It is clear that there are many biological adaptations for language including specialist enervation of the respiratory and vocal tract, tuning of hearing to the bandwidth of speech, the auditory-vocalization pathways for vocal imitation, neural adaptations like slight extension of the arcuate fasciculus [5], and genetic adaptations like variants of FOXP2 which facilitate language production through developmental pathways [6]. Yet it is also clear that the great bulk of language complexity, by contrast, must be attributed not to an innate source but to cultural evolution, for languages differ in their construction on every level, from the sounds to the syntax. Indeed, apart from the complexity of human communication systems, the thing that sets language apart is its sheer diversity across human groups—no other animal (as far as we know) has a communication system which can differ so fundamentally in both sound, form and meaning [7]. As Darwin [1, p. 56] remarked, 'language is an art made possible by an instinct to learn'.

This diversity raises a puzzle about how the physiological adaptations could have evolved to support such a moving target. One answer to this puzzle is to appreciate the central contribution that human interactional capacities make to language, since in contrast to the diversity of languages these interactional characteristics turn out to be nearly uniform across the species. For example, there are precise parallels across languages and cultures in the temporal properties of turn-taking in conversation [8], in the mechanisms that maintain intersubjective understanding through ‘repair’ of unclear utterances [9], and in the organization of sequences of contingent responses [10]. There therefore appears to be a much greater constancy in the niche for language usage and language learning than in the organization of languages themselves. It is this interactional niche and the properties characterizing language use which has probably played an instrumental role as a crucial constant target for the biological adaptations underpinning language: human interactional abilities offer a ‘machine tool’ as it were for producing languages.

Darwin's adversary Max Müller [11, p. 403] famously held that ‘language is our Rubicon, and no brute will dare to cross it’. However, when attention is turned to the underlying interactional abilities that make language possible, continuities between human and other primate communication systems become much more apparent. Human languages have evolved in a quite specific communicational niche—a highly intensive form of social interaction with special properties—the roots of which however can be seen in other primates (e.g. [12,13]).

## 2. The ‘interaction engine’

On this account then, human languages have evolved and diversified though cultural evolution, being learned by each successive generation. This makes the context for acquisition by children crucial. The prime ecological niche for language in general and language acquisition in particular is social interaction—this is the context in which languages are learnt by children, using native interactional capacities which allow them to bootstrap themselves into their local tongues. Some time ago, I outlined the case [14] for a specific type of human social interaction that is not itself linguistic, but which makes language possible. The capacities that enable this form of communicative interaction I dubbed the ‘interaction engine’, including crucially the attribution of intention to communicative acts involving a modelling of the other's mentation, and even a modelling of the other's model of ego's intentions. This last is what makes it possible to invent a signal, as when I rub my chin to confidentially indicate you have egg on yours (my signal is based on a Gricean intention to get you to wonder what I must be trying to get you to think). In addition, I point to a bunch of more easily observable features that characterize human communicative interaction, including turn-taking, contingent sequences and multimodal signals.

In the succeeding years, a great deal of research has borne out those original suggestions. Large spoken corpora in numerous languages have established striking uniformities in conversational behaviour [8–10], starkly contrasting with further evidence for structural diversity across languages [15]. Experimental and neurocognitive work reveals the extraordinary work-arounds that make linguistic processing possible in the tight timing of conversational exchanges [16,17], while work on infant–caretaker interaction shows that ‘proto-conversation’ is early established in the first year of life [18–20], and is evidenced in the most remote societies [21]. Work on autism spectrum disorders provides evidence that many aspects of the interaction engine are systematically disrupted together [22]. One of the most exciting developments has been the exploration of many of these properties across the primate order, suggesting precursor abilities in many other species, e.g. communicative turn-taking [23–25].

Here we focus on some principal components of the ‘interaction engine’ ([26]; for a more comprehensive list of design features see [27]), namely: (i) orchestration of multimodal signals (gesture, vocalization, gaze, facial displays), (ii) highly controlled timing (e.g. tight response latencies), (iii) manipulation of specific contingencies between initiating signal and response, and (iv) aspects of what has been called ‘theory of mind’, specifically the ability to attribute detailed intentions to communicative acts.

Taking these four components in turn, we here sketch the human specificities of each. First, then, multimodality: given the pre-eminence of speech it is easy to overlook the fact that language is primarily produced within a multimodal display, with gaze to addressee, facial displays, and near obligate gesture of the hands [28]. There are very interesting questions, but few answers, about how these behavioural streams are coordinated and understood—parts of the behavioural stream that are understood to belong to one another are often not synchronous [29]. In general, the multimodal nature of animal communication is also often underplayed [30]. As Darwin [31] noted in the context of expressions of emotion, there are clear phylogenetic continuities here with other primates, but also (as more recently observed) some human specialisms like the white sclera that makes human gaze so trackable [32].

The second component is timing. Human communication is typically done in alternation, taking turns at short bursts of communication, averaging about 2 s long, with very rapid transitions of the order of 200 ms between turns [17]. These timings are very similar across languages and cultures [8]. The response timing remains more or less the same in sign languages [33], which is interesting because it shows that the alternation of turns is not motivated by avoiding masking of the auditory signal (signs are produced by the motor system but perceived by the visual system with no interference). There is great sensitivity in timing, as slight delays are interpreted as meaningful [34]. Again, phylogenetic precursors are clearly in evidence, as vocal turn-taking systems can be found scattered right across the primate order, usually with slower turn-transitions around 1 s long ([23]; fast turn-taking is especially prominent in the Callitrichidae, notable for their alloparenting, relevant below). The great apes seem to be the exceptions as vocal turn-taking is not in much evidence, but their more flexible gestural communicative systems are remarkable for showing timing characteristics (e.g. 200 ms response intervals) very close to the human norm [24]. Given the great ape evidence, a popular theory of language evolution suggests an early gestural phase, and this is consistent with palaeontological evidence which suggests that the breathing adaptations for speech post-date early forms of African *Homo erectus* [35].

The third component is the set of contingencies between a turn and its response. In language usage, this is the relationship between question and answer, request and compliance, greeting and reciprocal greeting and so on. In humans, these are highly elaborated in two directions: first, there is a huge inventory of such ‘speech acts’ and the potential to always invent more, and second, there is the indefinite recursive elaboration of these sequences. For example, after a question another question–answer pair may intervene before the answer is supplied, as in (from [36, p. 333]):


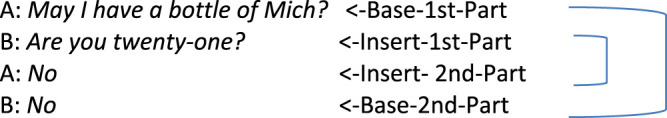


Indeed, since far deeper centre-embedding is found in these conversational sequences than in natural language grammar, this is a possible origin for the syntax of non-local dependencies [37]. In any animal communication system, contingencies between signal and response are the indication that some kind of communication has effectively taken place, and detailed contingencies have been observed both in monkey call exchanges (see [38]) and in ape gesture systems, e.g. with request-compliance exchanges between chimpanzee or orangutan infants and mothers [39].

The final component discussed here is the ability to accurately attribute intentions to communicative turns, involving at least some aspects of what has been called ‘theory of mind’. For the contingencies just outlined hold, not mechanically between the form of the utterances in human communication, but between their underlying intents, actions or purposes. For example, ‘It's six o'clock’ could serve variously as an excuse to leave, a suggestion to go to the pub, the declaring of a meeting open, and so forth, according to sequence and context. It is this feature that above all gives human communication its flexibility. It presupposes basic theory of mind—that actions are explained by goal-driven purposeful behaviour. However, more than that, it requires grasping the idea that an action has been done specifically to have the intention recognized, for that is its sole or main purpose. I can push the wine bottle towards you, suggesting you have some more, or I can make as to fasten a button suggesting you do up yours. Actions that might look like practical actions can be done in such a way as to indicate they are actually communicative, as when I rub my chin vigorously to indicate you still have breakfast on yours. The successful attribution of communicative intentions is dependent on assumptions of veridical (or at least helpful) communication, cooperation and trust.

These four components (multimodality, timing, contingency, intentionality) are the critical design features that make human communicative interaction possible. Studies comparing their properties across a dozen or more human language and cultures, mostly unrelated, indicate that these features are universal, in the sense that they are relatively uniform across unrelated cultures.

Comparative ethology across humans and primates suggests that these components were added cumulatively over deep time, and before vocal language as we know it. Fully vocal language probably developed somewhere after early forms of *Homo erectus* (who seems to have lacked the voluntary breath control essential to fine speech production) but before the last common ancestor of modern humans and Neanderthals, so between say 1.4 Ma and 700 Ka [40]. However, early tool using (and certainly Mode II or Acheulian technologies) suggests some advanced form of communication afforded cultural and technological transmission far earlier [41]. If so, it must have been at least partly gestural given the lack of respiratory control in African *Homo erectus* [35], and was plausibly built on the antecedent ape gesture system, so inheriting the turn-taking timing shared between humans and chimpanzees. That language usage has developed within an antecedent turn timing straightjacket is suggested by the severe cognitive processing challenges that now have to be overcome both in child language acquisition and adult production, where complex syntax is squeezed rapidly into short turns [17]. Given such a gradual accumulation, it is legitimate to ask whether these four components (multimodality, timing, contingency, intentionality) form the core of an integrated system. Here evidence from autism spectrum disorder (ADS) suggests they do, since ADS often exhibits simultaneous impairments of gaze, turn-taking, timing [42] and gesture [43], sequencing of contingent acts and ascription of intentions [44].

As pointed out, each of these interactional components have clear precursors in the communicative behaviour of other primate species [23,45], but the highly developed intentionality of human communication still seems quite exceptional. Much discussion of language treats it on a coding analogy: speakers code their thoughts in the conventions of their language, and hearers knowing the conventions decode the intended thoughts. This is how computers communicate with one another, but not how human language works. Human language capacities rest squarely on the ability to infer communicative intentions. Consider the following exchange where no compliment is actually encoded:

A: *I could eat the whole of that cake*.
B: *Thanks. It's quite easy to make actually*.

Intention attribution also serves to unlock novel expressions like ‘nano bonus’ or ‘muscular speech’. Linguists point out that nearly every ordinary utterance relies heavily on disambiguation and pragmatic resolution based on inferences about speaker's meanings or intents (‘that is a good point’ could be appraisal of the knife in your hand, or appreciation of the argument you just made). Even the simplest indexical pointing of the hand requires resolution (is what is indicated an object, its colour, the desire for it, a direction, or what?). All these inferences to the best interpretation rely on the assumption of cooperative communication, together with rules of thumb that follow from that assumption [46]. The language learning situation relies on the child grasping the intentionality behind utterances—even the learning of a name or referring expression requires inferring whether the indicated thing is a type or token, a quality or an entity. The point then is this: it is not language that has made human social interaction possible, it is rather our interactional abilities that have enabled language, and a crucial ingredient is this rich intentionality, this reflexive mind-reading. The question then arises what exactly is the evolutionary background behind this crucial ingredient in human communicative interaction. This question is the focus of rest of this paper.

## 3. The origins of intention recognition in communication: ‘cuteness selection’ and alloparenting

In exploring how the intention-recognition capacity of the ‘interaction engine’ may have evolved, one naturally looks for precursors among the other primates, specifically one looks for behavioural niches where empathy and ‘theory of mind’ might be exhibited. Although apes clearly grieve for their dead, there is no evidence that non-human primates show empathy in cases other than distress [47], and apes at least do not seem motivated by sympathy to perform pro-social acts [48].

An important and unresolved question is whether the non-human great apes exhibit any signs of complex reflexive intentionality. First-order intentionality (attempts to get the other to do something) does not seem in doubt, but second-order intentionality (attempts to get the other to think or desire something) and beyond remains controversial, despite evidence for persistence, repair, upgrading and audience-design [30]. Critical will be evidence about how the gesture signals, the characteristic flexible communication systems of the great apes, actually get acquired. Some theories hold there is an innate repertoire [49], others that many gestures are interactionally learnt ([50]; see also [51,52]). The latter would open up the possibility of third-order intentionality (the attempt to get the other to think such-and-such by virtue of the recognition of that attempt, i.e. full Gricean ‘meaning’), a possibility that Dennett [53] has argued should at least be entertained to see if it yields interesting predictions (and see [54]).

However, one domain where most mammals and not just the great apes tend to display interest in, and caring for the mental and physical states of others, is the maternal relationship between mothers and offspring. This is the locus for a restricted and specialized ‘theory of mind’, as required for intermittent on-demand feeding and other kinds of infant care. It is notable that this maternal relationship is the environment in which a flexible gesture system first gets established in the development of chimpanzees [24,50], just as it is the key setting for the first stages of language acquisition (in both cases of course other social partners play a key role in the full development of the communicative repertoire; [50]).

Now, humans are clearly an alloparenting species, that is, they engage carers other than the mother alone in the child-rearing process. Alloparenting frees the mother for further reproduction, and in fact humans in traditional societies have interbirth intervals close to half those of the other apes (around 29 months in humans versus around 60 months in chimpanzees), and early adoption of shared parenting must have been a key factor in human demographic success [55,56]. We are in effect the rabbits among the apes. By contrast, alloparenting is largely precluded in chimpanzees by the risk of infanticide [47]—chimpanzee mothers jealously guard their infants, holding onto them even when they have died. Consequently, chimpanzee twins rarely survive outside captivity [57].

Alloparenting requires a generalization of the maternal relationship, with the mother's interest in the infant's needs emulated by other adults or older offspring [56]. It also incidentally requires community-wide generalization of communicative signals—ritualized signals developed inside the maternal relationship will not be enough. The evolutionary mechanisms for such a generalization of aspects of theory of mind may have been various, but one possibility is a mechanism that has not been much discussed, namely what can be called ‘cuteness selection’ [58]. ‘Cuteness selection’ is the evolutionary mechanism coupling appealing features and caring responses. Konrad Lorenz first identified the human preference for the large eyes, globular skull, reduced jaw, chubbiness, high-pitched vocalizations and so forth both in offspring and our pets ([59]; see also [60,61]). The deep almost irresistible appeal of cute features to humans is commercially exploited in the pet trade, the comic industry whether Disney or manga [62], and in artistic kitsch [63].

This match between stimulus (cuteness) and response (caring) may have been originally driven by the same mechanisms identified by RA Fisher under the rubric of runaway sexual selection [64]—applied not only to mate selection but also to infant survival success. Fisher's target was the handicap feature like the peacock's tail which caused Darwin such sleepless nights: what started off as an honest signal of fitness, once locked into an automated stimulus–response cycle, can accumulate to a degree where it is actually detrimental. Such an originally virtuous cycle may lie behind the irresistible attraction of ‘cuteness’ features: if parents invest more in ‘cuter’ offspring, their own preferences may be passed on as well as the stimulus that triggers the preference; similarly, if mates by this mechanism come to prefer ‘cuter’ mates, the process can accelerate. At the same time, alloparenting reduces the mother's investment, so that human mothers are more likely to abandon infants than chimpanzee mothers [56]. Thus ‘cuteness’ becomes an important counterweight to possible abandonment in times of stress, so reinforcing the fitness benefits of ‘cute’ features. Importantly, along with the external ‘cuteness’ stimuli go the caring response instincts, the empathy and the interest in emotional states it triggers. Alloparenting would greatly increase the fitness benefits of both the ‘cuteness’ stimulus and the caring response, and motivate the generalization of maternal instincts outside the maternal relationship. In this way, ‘cuteness selection’ offers a feedback process, similar to Fisher's runaway sexual selection, that could have generated the overall generalization of empathy and ‘theory of mind’ that characterizes our species.

If attending to an infant's needs and desires involves first-order theory of mind (a model of the other), trying to reassure, console, or express caring and affection can involve third-order theory of mind, namely a model of what the other will make of those signals. It is this third-order that is essential to the flexible use of language, the narrowing of reference and the use of novel expressions—i.e. the level of Gricean intentions. When I rub my chin vigorously intending you to think you may have egg on yours, you decode it by recognizing a non-instrumental action and wondering what led me to produce it. Since ape gestures (like human utterances) seem sometimes novel [52], and typically vague or ambiguous, they have to be resolved in context—suggesting that these systems already involve higher order intentionality [65]. It is this possibility for constructing signals that allows a language to grow, and indeed to function by pragmatically fleshing out the meaning. While higher order intentionality does not alone account for language, it is a precondition, and with the incremental growth of a conventional system of symbols higher level intentionality is itself reinforced [66]. Being able to express thoughts about others' states of mind will certainly enrich theories of mind, as studies of emerging sign languages have shown [67]. This ‘maternalization’ of the general population through ‘cuteness selection’ may not only have sown the seeds for language but may also help to explain the default trust and cooperation that is found inside (but not necessarily outside) human and other alloparenting groups [68]. In addition, ‘cuteness selection’ may offer a rival theory to the self-domestication theory recently invoked to explain cranial globuralization and associated changes in the human lineage [69]. Researchers have pointed to many features of human evolution (including gene expression) that appear neotenic (paedomorphic), or more exactly heterochronic in origin, including extended childhood [70,71]. Traditionally, the cranial aspects of these processes have been attributed to reduction of the dentition consequent on dietary changes and to fire. Recently, though, it has been suggested that the different cranial shapes of modern humans and Neanderthals can be attributed to ‘self domestication’, wherein a reduction of aggression alone carried with it a host of other features, as has been noted in domesticated animals [72]. There are problems with this theory though (see also [73]). Firstly, domestication is typically associated with drastic reduction of the brain (in pigs up to 35%; [74]), and although early modern humans show a small reduction from Neanderthal specimens, the reduction is in proportion to differences in body mass; generally, and over the much longer period, human evolution is of course associated with massive brain increases. Secondly, domestication is associated with an all-round loss of aggression (for that is the major target of domestication), but humans are one of the most aggressive of all mammalian species, it is simply that the aggression is typically outgroup directed, and is mostly premeditated and collective [75]. ‘Cuteness selection’ offers an account of the trend to human ‘neoteny’, anatomical gracialization and extended childhood that does not have these difficulties and would have worked on the much longer timescale of hominin evolution over the last couple of million years. That time scale matches recent endocranial studies showing growth in language-related brain areas separating earlier *Homo erectus* specimens pre-1.5 Ma from later ones [76], which is in line with the likely (but still controversial) possession of full language capacities by the common ancestor of Neanderthals and modern humans by ca. 700 Ka [40]. By contrast, the self-domestication thesis would link language to post-Neanderthal brain reduction, while the palaeontological and genetic evidence so far points to a pre-Neanderthal date for extensive spoken language [3,40].

One of the proximal effects of ‘cuteness’ is that it releases oxytocin in the recipients, a hormone that plays a role in many physiological processes, being upregulated in pregnancy and lactation. Its role in human and animal bonding has been much studied: it reduces fear, enhances trust and promotes prosociality [77]. It increases empathy and encourages gaze at interactants' faces [78], and reduced levels are associated with autism and other interactional deficits [79]. Different levels of the hormone are associated with the greater sociality (empathy, tolerance, play) of bonobos compared to chimpanzees [80,81], and it and other endogenous opioids are upregulated in humans compared to apes [82]. Especially relevant for the current argument is that administration of oxytocin enhances communication and the effective targeting of messages [83]. It thus seems plausible that runaway cuteness selection could have driven the generalized ‘theory of mind’ essential to the way modern human language works.

The account outlined here and in [58] emerging from linguistics and anthropology converges with that given by Hrdy & Burkart [84] based on data from primatology. Both accounts see causal connections between alloparenting and theory of mind, but the Hrdy & Burkart account emphasizes the infant's role in beguiling and manipulating reluctant care-takers, while the present theory puts the emphasis on adaptations in empathy and caring in the alloparents, triggered by cuteness features. Neither theory explains why other primate alloparenting species, largely among the callitrichids, show little evidence of theory of mind; both theories must assume that the large-brained human ancestors were fertile ground, or that (as in the present theory) a runaway process of cuteness selection happened not to be impeded by other physiological requirements like sexual dimorphism or a diet requiring massive dentition.

## 4. Human interaction and human social systems

Human social life is held together by intense social interaction. It has been estimated that humans spend up to 30% of waking hours in such interaction, compared to perhaps 20% among chimpanzees [85], although in both cases there are large differences across particular social groups. Fire must have greatly extended the social day and enhanced interactional opportunities [86]. A cross-cultural study of conversation suggests that individuals spend on average 4.5 h conversing, in which time they each may produce 16 000 words in 1500 turns at talking (extrapolating from [87]).

What drives the extraordinary human investment in interaction? Alloparenting, the outsourcing of infant care, depends on building bonds within social groups, and such bonds would also have been crucial in the earliest phases of hominin cooperative scavenging and hunting [88,89]. The multiple childhood attachments offered by alloparenting would have facilitated the acquisition of skills and information crucial to building cultural adaptations. There were probably multiple incentives of this sort for investing in interactional partners. In addition, Dunbar has suggested that language, because of its broadcast character, arose as a form of mass grooming, so knitting together large groups. However, this neglects both the fact that chimpanzees often groom in large chains [90], and that human conversational huddles are mostly in the twos or threes. However, pointing to the primary social functions of language is surely correct. Studies suggest that around two-thirds of what is discussed in informal conversation concerns social relationships [86,91]. The central role that social navigation plays in human social life is reflected in the way languages have elaborated conventions for signalling social relationships, for example, the elaborate honorifics of Japanese or Javanese, or in English the many indirect request forms (*Can I…, Could I possibly…, Would it by any chance be possible…*etc.) which achieve the same sort of effect. One of the central functions of language is thus the delicate juggling of highly sensitive social relationships. We have inherited from the common ancestor with our nearest primate relatives a fission–fusion society where relationships have to be constantly negotiated. In the pre-agrarian bulk of human history, this fluid social structure allowed conflict to be resolved simply by decamping, or else escalated by joining forces with kinsmen. In addition, humans in greatly elaborating divisions of labour and domains of expertise have engineered a system where social relationships are situationally dependent—I may be the nautical expert, but you the expert curer, orator, hunter or fisher: who leads depends on the activity. Conversation analysts have shown that these domains are actually jealously guarded and defended by the details of language use [92]. Even in the midst of the most rigid human social systems (rural caste India for example), social relationships are under constant juggling. The flexibility of human communication systems owe much to the fluidity of human social relationships and the social structures built out of them.

## 5. Conclusion

This paper has argued that human language rests on an infrastructure for communicative interaction. It also of course relies on many anatomical and neurocognitive adaptations for language, but the evolutionary launch pad for those features depends on the prior acquisition of the interactional infrastructure that would have made language both possible and adaptive. Precursors for many aspects of this interactional infrastructure (turn-taking timing, contingent signals, multimodality) can be seen in other primates, but despite precursors, the evolution of full-blown intention recognition and theory of mind seems more puzzling. Here we have argued that one possible account for how humans developed the extended ‘mind-reading’ that makes language feasible is based on the generalization of mother–infant sensitivities, spurred by alloparenting and the possible runaway characteristics of ‘cuteness selection’.

## Data Availability

This article has no additional data.
